# Eczema Capitis

**Published:** 1874-05

**Authors:** 


*Eczema Capitis.*—Cure if possible, notwithstanding the somewhat prevalent idea to the contrary. The method of procedure recommended is as follows:

First, apply a poultice every night until all the scabs are removed. The ulcerations, which are sometimes present after the scabs have been removed, are best cured by the application of a wash made of nitrate of silver grs. v to the ℥ i of water. The following is then used with good success:

| | | |
|---|---|---|
| ℞.—Aquæ Cologn, . . . . . | ℥ iv. | |
| Glycerine, . . . . . . . | ℥ ij. | |
| Carbolic acid crystals, . . . . | ʒ j. | |
| Borax, . . . . . . . . | ʒ j. | M. |

Continue the application of this remedy for some time for the purpose of curing the eczema.

